# The Disruption of Trust in the Digital Transformation Leading to Health 4.0

**DOI:** 10.3389/fdgth.2022.815573

**Published:** 2022-03-28

**Authors:** Michael Guckert, Kristina Milanovic, Jennifer Hannig, David Simon, Tamara Wettengl, Daniel Evers, Arnd Kleyer, Till Keller, Jeremy Pitt

**Affiliations:** ^1^Cognitive Information Systems, KITE-Kompetenzzentrum für Informationstechnologie, Technische Hochschule Mittelhessen-University of Applied Science, Friedberg, Germany; ^2^Department of MND-Mathematik, Naturwissenschaften und Datenverarbeitung, Technische Hochschule Mittelhessen-University of Applied Science, Friedberg, Germany; ^3^Department of Electrical and Electronic Engineering, Imperial College London, London, United Kingdom; ^4^Department of Internal Medicine 3-Rheumatology and Immunology, Friedrich-Alexander University (FAU) Erlangen-Nürnberg and Universitätsklinikum Erlangen, Erlangen, Germany; ^5^Lilly Deutschland GmbH, Bad Homburg, Germany; ^6^Department of Internal Medicine I, Cardiology, Justus-Liebig-University Gießen, Gießen, Germany

**Keywords:** Health 4.0, trust, artificial intelligence, medical education, supervised exercise, virtual reality, public health

## Abstract

The specification and application of policies and guidelines for public health, medical education and training, and screening programmes for preventative medicine are all predicated on trust relationships between medical authorities, health practitioners and patients. These relationships are in turn predicated on a verbal contract that is over two thousand years old. The impact of information and communication technology (ICT), underpinning Health 4.0, has the potential to disrupt this analog relationship in several dimensions; but it also presents an opportunity to strengthen it, and so to increase the take-up and effectiveness of new policies. This paper develops an analytic framework for the trust relationships in Health 4.0, and through three use cases, assesses a medical policy, the introduction of a new technology, and the implications of that technology for the trust relationships. We integrate this assessment in a set of actionable recommendations, in particular that the trust framework should be part of the design methodology for developing and deploying medical applications. In a concluding discussion, we advocate that, in a post-pandemic world, IT to support policies and programmes to address widespread socio-medical problems with mental health, long Covid, physical inactivity and vaccine misinformation will be essential, and for that, strong trust relationships between all the stakeholders are absolutely critical.

## Introduction

Health 4.0 (1) is a term that has been coined for the trend that transfers general principles of Industry 4.0 (2) to the health care domain. Industry 4.0 addresses the increasing application of advanced ICT (Information & Communication Technologies), data science and AI (artificial intelligence, especially machine learning) in manufacturing, factory automation, supply chain management and logistics, and the “4.0” derives from three preceding revolutions. While Health 4.0 is intended to align with the use of ICT, data science and AI in medical applications and education, individual diagnosis and therapy, and public health, three preceding revolutions can also be identified [cf. (3)]:

- *Health 1.0*, following an era of superstition and belief in witchcraft, covering the period from the Hippocratic Oath (5th century BCE), to the Renaissance (1600 CE), including systematic training of physicians and documentation of treatments and interventions;
- *Health 2.0*, the basis of modernity, including the germ theory of disease, and the discoveries of vaccination and antibiotics; and
- *Health 3.0*, or “modern” medicine, i.e., evidence-based medicine using systematic reviews and meta-analysis, a deep understanding of the natural sciences in pharmaceuticals, physical instrumentation, and chemical treatment and an emphasis on public health with respective heath policy as a common good.

Of particular interest here is the Hippocratic Oath, which codified various ideals of professional medical practice. Over time, many educational institutions have chosen their own rendering. For example, at graduation, the University of Sheffield Medical School asks its graduates to participate in the recitation of *The Sheffield Affirmation* (4), of which one clause is:

*I will, in the course of my work, come into special relationships with my fellow human beings, calling for great propriety and trust. I will avoid all wrong-doing and anything mischievious or dishonorable*.

This requirement of both ***propriety*** and ***trust***, on the part and practice of the health practitioner, warrants deep consideration in the context of specifying and applying policies and guidelines in the context of Health 4.0. This is because technological innovations, which can improve diagnostic and therapeutic instruments, also change the role of the doctor and her trust relationship with the patient. Moreover, these innovations have a profound impact on other stakeholders of current health systems: Health 4.0 comes with a shift that makes some companies become service providers instead of product manufacturers, and possibly have direct patient interaction. Consequently, the trust relationships between the various stakeholders–regulatory authorities, medical practitioners and patient themselves–has been made more complicated, but also potentially compromised, by the introduction of new players. Great propriety is something a medical practitioner must do, but great trust is something a medical practitioner must *get*–by acting with great propriety.

Trust has a long history of scientific discourse and has been subject of discussions in and between various disciplines, including formal models from computational perspectives (5). Trust in the social world subsumes the expectation of cooperation, support, and a principally non-hostile behavior of participants of social interaction in the widest sense, while in the interaction with machines and technical devices, trust can be viewed as the expectation that consented features of the device will reliably, i.e. with high probability, work as defined even in the presence of externally induced uncertainty (6). Trust constructs a mutual relation which always implies a willingness on at least one side of the relation to accept vulnerability and to sacrifice control and increase dependency (7). While we may assume a principal credence in the health system based on experience and tradition we now face changes induced by the introduction of technology.

The primary goal of this paper is to shed light on the impact and potential shifts of the health ecosystem induced by technological innovations. We therefore concentrate less on what trust “is,” as widely discussed in the prior literature [e.g., (5)]. and focus instead, firstly, on what trust “does” (i.e., coordinate expectations and short-cut the complexity of decision-making in collective action situations characterized by uncertainty); and secondly, on the potential *disruption* of this socially-constructed relationship caused by the introduction of new stakeholders in the ecosystem, some of whom may not be incentivised to act in good faith by other normative social constructs like contracts, treaties or, as particularly concerns us here, credible pre-commitments (e.g., the Hippocratic Oath).

The problem addressed in this discussion of trust in the healthcare system is then analogous to e-commerce and the use of distributed consensus technologies in commercial transactions: the technology provider is leveraging a pre-existing, and well-established, trust relationship (between people and vendor, banks, etc.), and asking the people to trust the technology/programmers instead–yet this is by no means assured. Similarly, the technology providers behind medical applications in e-health or Health 4.0 are, in effect, leveraging the pre-existing, and well-established trust relationship between individuals on the one hand, and their doctors, nurses, and surgeons on the other, and transferring it to the technology (and its programmers) instead. Yet neither of these have taken (or even could take), the Hippocratic Oath, the Sheffield Affirmation, or other equivalent commitment to professional practice, i.e., being loyal to the responsibilities of the profession, refrain from any action which may be harmful, respect confidentiality, or have a “special relationship with fellow human beings.”

Therefore, we contend that the specification and application of policies and guidelines for public health, medical education and training, and screening programmes for preventative medicine are all predicated on well-established trust relationships between medical authorities, health practitioners and patients. These trust relationships are in turn founded on (some variation of) a verbal contract that is over two thousand years old. Our concern is that the impact of ICT underpinning Health 4.0 has the potential to disrupt this analog relationship in several dimensions; but our hope is that it also presents an opportunity to strengthen it, and so to increase the uptake and effectiveness of new policies. The aim of this paper is to address that concern, and help turn that hope into reality.

This paper shows how to take advantage of this opportunity, and is structured as follows. Section Analytic Framework for Trust Relationships in Health 4.0 presents a general analytic framework for the trust relationships in Heath 4.0. Section Use Cases examines in detail three use cases, each of which assesses a medical policy, the introduction of a new technology, and the implications of that policy-technology binary for the trust relationships. We integrate this assessment in section Implications and Actionable Recommendations in a set of actionable recommendations, in particular that the trust framework should be part of the design methodology for developing and deploying medical applications. In a concluding discussion in section Discussion and Conclusions we advocate that, in a post-pandemic world, IT to support policies and programmes to address widespread socio-medical problems with mental health, long Covid, physical inactivity and vaccine misinformation will be essential, and for that, establishing strong, reliable and verifiable trust relationships between all the stakeholders is absolutely critical.

## Analytic Framework for Trust Relationships in Health 4.0

**Figure 1:**
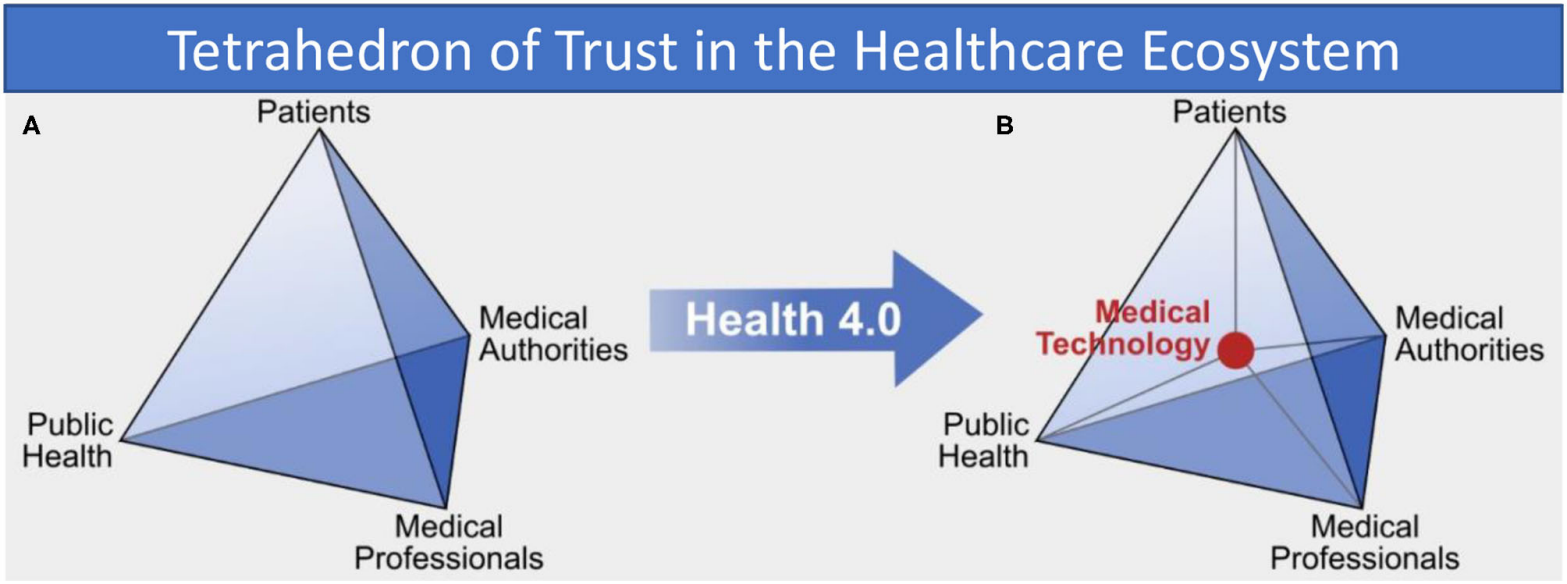
**(A)** Trust relations between “traditional” health system stakeholders and **(B)** in the Health 4.0 socio-technical ecosystem, showing how medical technology is an intermediary in all interactions.

Taking the perspective of public health as a kind of common-pool resource and applying Ostrom's approach to institutional arrangements for managing complex systems that are neither market-driven or state-controlled (8), we can identify four primary stakeholders in traditional (pre-Health 4.0) approaches. These four invested parties, as illustrated in Figure 1A, are patients and patient-representation groups, medical practitioners such as doctors and nurses, medical authorities which govern the practice of medicine (e.g., healthcare bodies like the National Health Service in the United Kingdom), and finally public health bodies such as governmental health departments and charities or lobby groups with particular healthcare interests (for example, diabetes). These four parties work together to shape and maintain each individual's health and the overall health of the population.

Applying the Jones (9) definition of trust from analytic philosophy (ordinarily, we say that *A* trusts *B* if (i) *A* believes there is a convention, norm or rule, and (ii) *A* expects *B*'s behavior to conform to that rule), we can specify how the four stakeholders in the health ecosystem need to construct and have established a mutual trust relationship with the others, as presented in Table 1. Patients, for example, trust both the medical practitioners who they consult directly, as well as public health and its institutions, e.g., the government or charities, to inform them according to scientific evidence and to principally have benevolent intentions. Health professionals trust their patients to act honestly during consultations and to follow the advice and instructions prescribed to alleviate or remedy their symptoms, i.e., the doctors believe there is a norm, e.g., “if a course of antibiotics is prescribed, then the patient will take the entire course,” and expects a patient to do exactly that (unfortunately, of course, the recent increase in anti-biotic resistant bacteria can be attributed in part to a breakdown in this trust relationship: doctors handing out anti-biotics like smarties and ill-informed patients not completing the course). Health professionals also have trust in their governing bodies, the medical authorities, to have the good of patients and society as a guiding principle. Medical authorities again trust that medical professionals are following their recommendations and communicate relevant findings in time and appropriate quality. This trust mesh, is also used within the health system to collect overall information about patients, diagnosis and therapies that can be monitored and aggregated into knowledge that is again spread through the ecosystem to improve healthcare. Underpinning this entire system is the age-old moral standard of doctors, the Hippocratic oath. Every party in this system trusts that they themselves and all the others uphold the ideals of doing no harm, respect confidentiality, and act with propriety (i.e., with respect to following or defining professional norms, standards, guidelines, policies and procedures).

**Table 1:** Health 3.0 trust matrix.

	**Medical practitioners**	**Patients and patient reps**	**Public health**	**Medical authorities**
Medical practitioners		Follow course of treatment	Allow diagnosis and treatment at individual level Respect for other perspective	Best practice, guidelines and professional safeguarding
Patients and patient reps	Objective diagnosis		Procedural justice best interests	Advice and information enforce rights of patients charter
Public health	Allow prevention and promotion at population level Respect for other perspective	Public Health 2.0 for information not misinformation		Be independent politically neutral perspective included in clinical practice
Medical authorities	Respect oath, follow guidelines	Respect responsibilities of patients charter	Be independent politically neutral	

The specific importance of trust relations for the health system as depicted in Table 1 and their fragility are not new. Reliability of individual clinicians and researchers are as important as the fundamental confidence of a society in its hospitals and clinics (10). Without a trustworthy stable environment of practitioners and medical institutions the overall trust in the health system is threatened to be undermined (11).

However, with the advent of Health 4.0 the traditional information exchange between trusted parties is changed fundamentally by the addition of technology. Analysis of exemplary interactions in the health system shows that medical technology now acts itself as an intermediary and takes a central position in the health system, as shown in Figure 1B. Technology and its developers now appear as new members of the healthcare socio-technical ecosystem. The introduction of new technology consequently adds a new class of stakeholder and so another strand of complexity to the trust relationships, but also introduces a number of new fragilities appears that all can potentially destabilize the entire trust network. The risk of unintended consequences of the use of technology and the immanent security threat of data collected by applications are two prominent examples.

Patients normally have trust in their general practitioners with whom they often share a long history of medical care. General practitioners themselves are accustomed to transferring patients to specialists when a disease requires more advanced diagnosis from a consultant. This requires trust in the skills of the specialist–at first from the general practitioners. The basic trust relation between general practitioners and patient is then projected onto the relation between consultant and patient. With the introduction of Health 4.0–analogously to that–doctors as well as patients must transfer their trust onto the devices and the AI algorithms. Doctors accept technical devices as additional instruments and expertise delivered by expert technicians. This requires trust in both the device with its algorithms and the engineers. Patients will use devices seriously and will have trust in the results if their GPs recommend it and consider in their diagnosis.

In summary, we observe that there is a socially-constructed conceptual relationship between different actors or stakeholders in the healthcare system which is conventionally labeled “trust.” There are definitions of health-system specific notions of the term trust that mirror this fact, e.g., seeing trust as the expectation that the trustee will act in a way of which the truster will approve, instead of the stronger and perhaps more reductive expectation that the trustee will always act in the interests of the truster (12). Furthermore, we see that the introduction of a new stakeholder in the healthcare ecosystem for Health 4.0 directly impacts this conceptual relationship and shows side-effects, with potentially both intended and unintended consequences which can be analyzed through the lens of this framework.

In the next section, we will analyse a set of representative use cases through this “lens” and identify the potential impact on the relations as described in Table 1. This will lead to an extended trust matrix for Health 4.0 in section Implications and Actionable Recommendations, from whose implications (section Implications) we derive a set of actionable recommendations (section Actionable Recommendations) for maintaining and building trust in Health 4.0.

## Use Cases

We present three use cases which embody examples for the beneficial use of technology in typical medical settings relating this use to relevant guidelines and policies. The first use case discusses a mobile application that gives patients suffering from intermittent claudication comprehensible and instructed access to physical exercise as is recommended but without support hardly viable for the patient. Ischemic heart disease and the diagnostic potential that lies in a comprehensive use of artificial intelligence-based algorithms possibly combined with smart devices are addressed in the second use case. Practical, case-based experience is an important cornerstone of medical education but hard to be guaranteed with necessary reference to real world patients when patients are strongly affected by the disease and suffer pain. After taking therapeutic and diagnostic perspectives in the first two use cases the last one therefore demonstrates how virtual reality can overcome this gap in education and training of doctors. Each case has implications with regard to guidelines and policies and raises trust as a crucial topic when technology and its providers enter the scene.

### Self-Supervised Exercise

#### Medical Policy

In the UK, there are over 700,000 people (1% of the population) suffering from the condition of *intermittent claudication*. Intermittent claudication is caused by an inadequate blood supply due to a narrowing or blockage of arteries in their legs, and patients may suffer from pain in their legs on walking, commonly in the calf (lower leg).

The UK NICE (National Institute for Health and Care Excellence) provides guidelines recommending supervised exercise programs: however, currently only one in four patients have access to such programs (13), and this has been restricted further by lockdowns limiting exercise.

However, vascular surgeons have recognized that the condition of many patients presenting with intermittent claudication and peripheral arterial disease is better treated by exercise rather than endovascular or surgical intervention (14, 15). Moreover, there are long-lasting benefits of supervised exercise over and above revascularisation, which include development of a social network, additional cardiac training, and motivational therapy (16). The initial problem is that this exercise causes pain, before and until the health improvements are realized; but patients experiencing pain tend to stop doing that which causes it–unless they are supervised performing the necessary exercise programs. Given the scale of the problem, supervised exercise can be an extremely costly and time-consuming use of medical resources–assuming that patients even have access to a hospital-based programme.

#### Technological Solution

**Figure 2:**
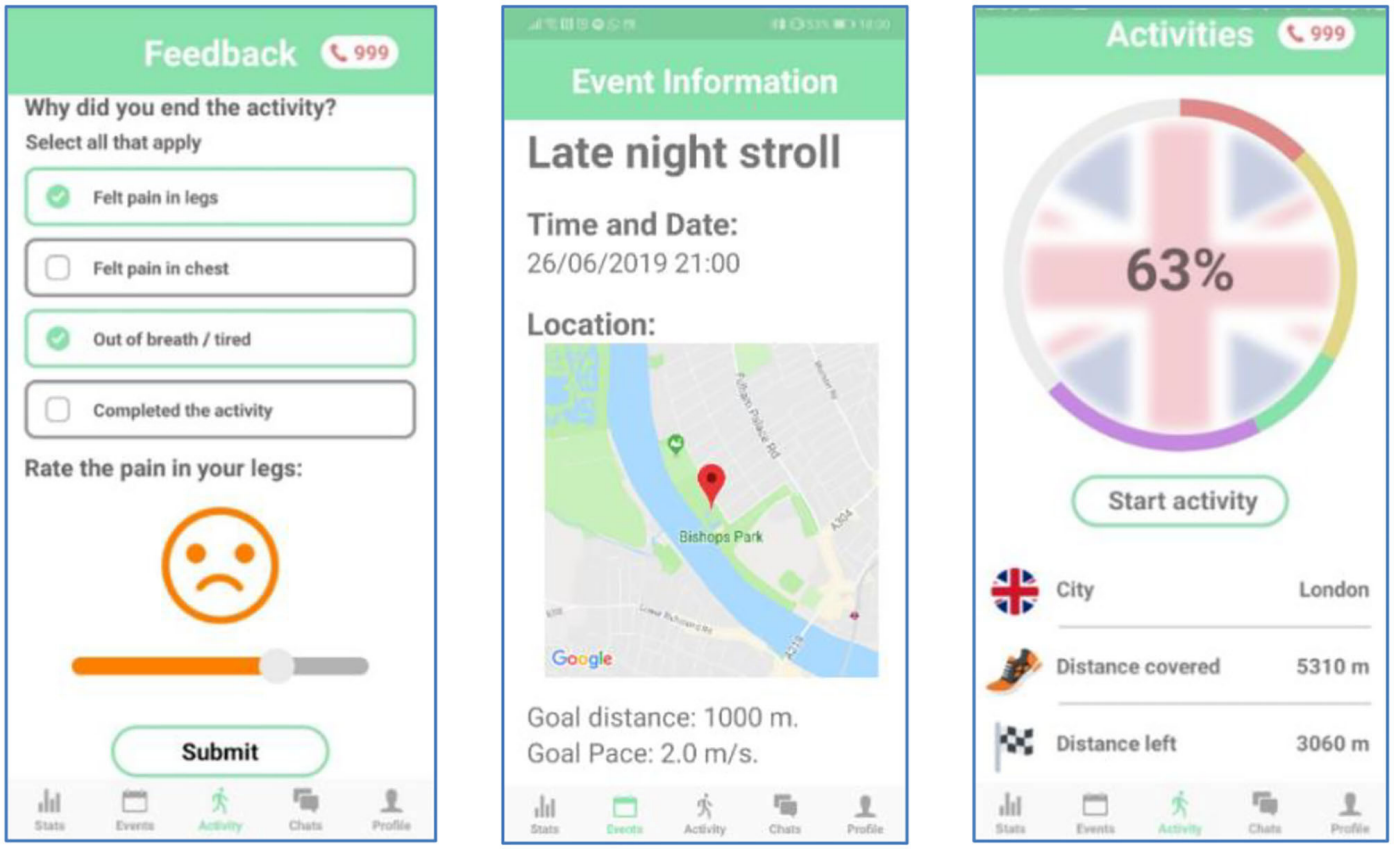
Screenshots from the ORBIS app.

ORBIS is a novel smartphone app which aims to bring about an improvement in public health on a national scale (sample screenshots of the app are shown Figure 2). The app aims to do this by decreasing the long-term impacts of intermittent claudication by increasing and encouraging participation. Generally speaking, apps that promote a ‘follower' mentality and lack remote supervision are less than ideal for targetting intermittent claudication in this manner. A follower mentality, coupled with an arbitrary reward system based on “kudos” is not desirable for a health application. It can encourage competition and incentivise unwanted behaviors: competition which could be helpful for some people, but it can demoralize others which can lead to self-regulatory failure (17). Moreover, some health apps are ostensibly concerned with health but are actually thinly veneered data collection programs which are focused on increasing profits for the app/service providers rather than the collective health of the users.

Instead, ORBIS promotes a “mutualist” approach in two ways: by enhancing and reinforcing relationships between medical practitioners and patients, and between patients and other patients.

Firstly, ORBIS digitally connects practitioners to patients, making remote-supervised exercise programs more accessible. Currently, remote supervision is not widely used and instead patients have to visit their doctor (or health practitioner) regularly or have a personal trainer dedicated to making sure they carry out their exercise regimes. In comparison to current methods, ORBIS reduces both time and monitory costs by implementing remote monitoring. It allows health practitioners to remotely enjoy economies of scale, saving the NHS (National Health Service) time, resources, and expenses. ORBIS acts as a green prescription by empowering patients, promoting collective action, and utilizing remote supervision to treat intermittent claudication.

Remote Supervision through the app offers a platform for the health practitioners to monitor and understand the progress made by their patients as a process, i.e., a trajectory rather than discrete steps in a point space. This provides the basis for prescribing new target distances and goals, rather than the medical practitioner having to be present supervising their patients during exercise, or making an assessment based on only a single session. This will allow practitioners to work with more patients at a time, and also to personalize the recommendations. This is achieved by providing the practitioner with all the information (e.g., distance covered, pace, number of breaks taken, etc.) related to each individual, for every event in which they take part. Practitioners can also provide feedback to patients using a chat interface.

Secondly, ORBIS digitally connects patients to other patients, making self-supervised exercise programs more accessible. The design of the app is motivated by some fundamental design concepts which aim to retain adherence rate among patients: these are self-supervision, gamification and collective action. We look at each of these concepts in turn.

Self-supervision is intended to encourage patients to walk longer distances, more frequently without the need of constant supervision from their doctor. Self-supervision mainly involves the individual assessing their own performance over time, and providing feedback to their health provider (see the “Feedback” screen of Figure 2), and this can be used to customize the exercise programme and as a motivation for improvement. This is facilitated by providing quantitative measures of their progress during an activity such as distance traveled, current pace, number of breaks taken, and inclusion of personal achievements. In addition, distance measurements over past events are visually illustrated using graphs, further encouraging patients to improve their absolute walking distance.

Constantly reminding patients about their symptoms can cause unwanted stress among patients. It can also cause compulsion. To encourage patients to walk frequently, and enjoy their experience, the app is branded as a participatory game rather than a mandatory chore. In the app, the users are immersed in a virtual “walk around the world” game, transforming their regular exercise routine into more of a leisurely activity. For example, the “late night stroll” of the “Event” screen of Figure 2 is used to inform the presentation on the “Activities” screen. In this way, as long walks through an entire city may seem daunting and discouraging to the user, each city is further broken down into iconic landmarks. As a result, the journey is viewed as a series of achievable “mini-goals,” increasing the likelihood that users will accomplish target distances. Finally, as users travel through the virtual cities, they discover walking related facts. This will act as an incentive for users to walk further with their groups, and consequently reach more destinations and uncover new facts.

Collective Action: Users exercise best when supervised, as they need someone to motivate them and overcome the pain they experience, in order to improve. Who are better to help with supervision, than patients who are in the same situation? The app should build communities of patients, that supervise each other and promote collaboration instead of competition. In the app, this is achieved by the use of the pie chart in the main activity page (see the “Activities” screen of Figure 2), which is broken down into different colored sections to represent each member's contribution. This should give rise to “healthy” peer pressure, encouraging less engaged members to walk further. Finally, patients will be able to chat with their group and organize events within the app. This not only promotes improvement with collaborative action but also brings about a positive impact on the quality of life of patients: where they have emotional support by having someone with the same condition with whom to socialize.

### ECG Diagnosis With Deep Learning Algorithms

#### Medical Policy

Doctors and medical professionals follow policies referred to as guidelines that contain recommendations for adequate diagnostic and best practice therapeutic measurements for diseases while balancing benefits and possible ensuing harms for the patient. Guidelines are published by professional associations in which committees of leading experts define standard procedures for known health issues in their respective sub-disciplines of medicine based on available scientific evidence. These issues range from diagnostic measures to preventive actions such as screening and medical checkups. Furthermore, guideline adherence often is associated with monetary regulations between e.g., health insurances and care providers. With regard to the presented use case and its affinity with the field of cardiology, national associations such as the German Association of Cariology (DGK; Deutsche Gesellschaft für Kardiologie) in Germany, the American College of Cardiology (ACC) in the United States, the National Institute for Health and Care Excellence (NICE) in the United Kingdom, or the European Society of Cardiology (ESC) on a European level just to name a few of the institutions, publish guidelines for practitioners in the field.

Ischemic heart disease (IHD) is among the most prevalent causes of death in countries of westerly lifestyle (18, 19), thus not only having a personal but also a high socio-economic impact. The disease strongly impairs life quality and causes high costs for both diagnosis and therapy (20). Presence of myocardial scar (MS) can be an indicator for IHD (21) and eventually might be a precursor of heart failure (22). Early detection of MS with high sensitivity would be of significant benefit, resulting in early therapy initiation and thus reducing individual suffering and costs on a health perspective level. High resolution cardiac imaging provides the information necessary to make appropriate therapeutic decisions (23) and, therefore, magnetic resonance imaging (MRI) is considered as a gold standard for non-invasive diagnosis of myocardial tissue conditions (24). Therefore, guidelines such as the ESC guideline for the diagnosis and treatment of acute and chronic heart failure, (25) recommend cardiac MRI using Late Gadolinium Enhancement for reliable diagnoses of MS.

However, there is only restricted availability and access to expensive high-resolution imaging medical equipment such as MRI scanners (26). Even in highly developed countries, the number of MRI scanners per citizen is limited [Germany: 33 devices / per 1 million inhabitants; USA: 36 devices / per 1 million inhabitants (27)]. As a result of these limitations and costs, MRI cannot be used as a comprehensive screening method on a population level for early detection of IHD (23), although it would be the method of choice from a medical perspective. The use of established, cost-effective, and almost ubiquitously available electro-cardiograms (ECG) for the early diagnose of IHD would be a potential feasible and preferable alternative. Unfortunately, the ECG applied and assessed by medical professionals does not achieve sufficient sensitivity to be recommended as screening method (28–31). Nevertheless, it has been shown that MS does have significant impact on the ECG (32) underlining that the technology itself does have the needed diagnostic potential. However, patterns in the ECG related to MS are heterogeneous and unspecific, and therefore difficult to detect by medical professionals that are not highly specialized (32, 33).

#### Technological Solution

Artificial intelligence in the form of deep learning (DL) algorithms can help making expert knowledge available in the broad. By distilling cardiologist expertise through supervised training of a convolutional neural network medical applications can be established as assistance tools in diagnostic algorithms for e.g., general practitioners. Screening of patients with a relatively cheap method becomes an option without the need to overuse the limited resource of MRI scanners.

Standard 12-lead ECG data, when combined with an appropriate selection of additional clinical parameters as the input to a DL model, can already yield diagnostic sensitivities and specificities which predict the presence of myocardial scarring (sensitivity: 84.7%, specificity: 91.2%, area under the curve (AUC): 87.9%) (34). This qualifies it for use in clinical environments.

This clinical usability of decision support systems is further supported by data such as a recent systematic review and meta-analysis of Liu et al. (35) showing that the performance of DL-based diagnostics of medical images has been compared to physician-level accuracy. An equivalent diagnostic performance of DL-models to that of health-care professionals has been reported based on the evaluation of 14 studies.

However, doctors apparently produce less false positives but also less true positives than algorithmic diagnosis instruments (36). Computer aided diagnosis combines the complementary strengths of the doctor–e.g., higher specificity-with that of the assisting machine–e.g., higher sensitivity. Further support for the promising use of AI for semi-automated ECG analysis is given by a meta-analysis that confirmed the ability of AI to predict heart failure from standard 12-lead ECG signals (37).

Moreover, deep learning models can give insight into how and why a result was derived and thus become a means of knowledge transfer by which medical professional and especially those without specific ECG expertise can extend their capabilities. For this purpose, explainability and interpretability of deep learning approaches have to be further elaborated in the future (38). With such a digital assistant e.g., general practitioners get an additional source of knowledge and experience they can use.

With increasing availability of wearable devices that can produce high quality ECG recordings, e.g., smartphones, smartwatches, etc. completely new options for early detection of heart diseases arise. A combination of artificial intelligence algorithms and smart devices delivering data can serve as foundation for an efficient and economically reasonable screening method for MS and other diseases. The principal feasibility has already been proven for atrial fibrillation and smart watch data (39). While such devices are currently still rather used as health and fitness trackers they can become comprehensible diagnostic means prescribed even by general practitioners to patients with specific risk factors.

**Figure 3:**
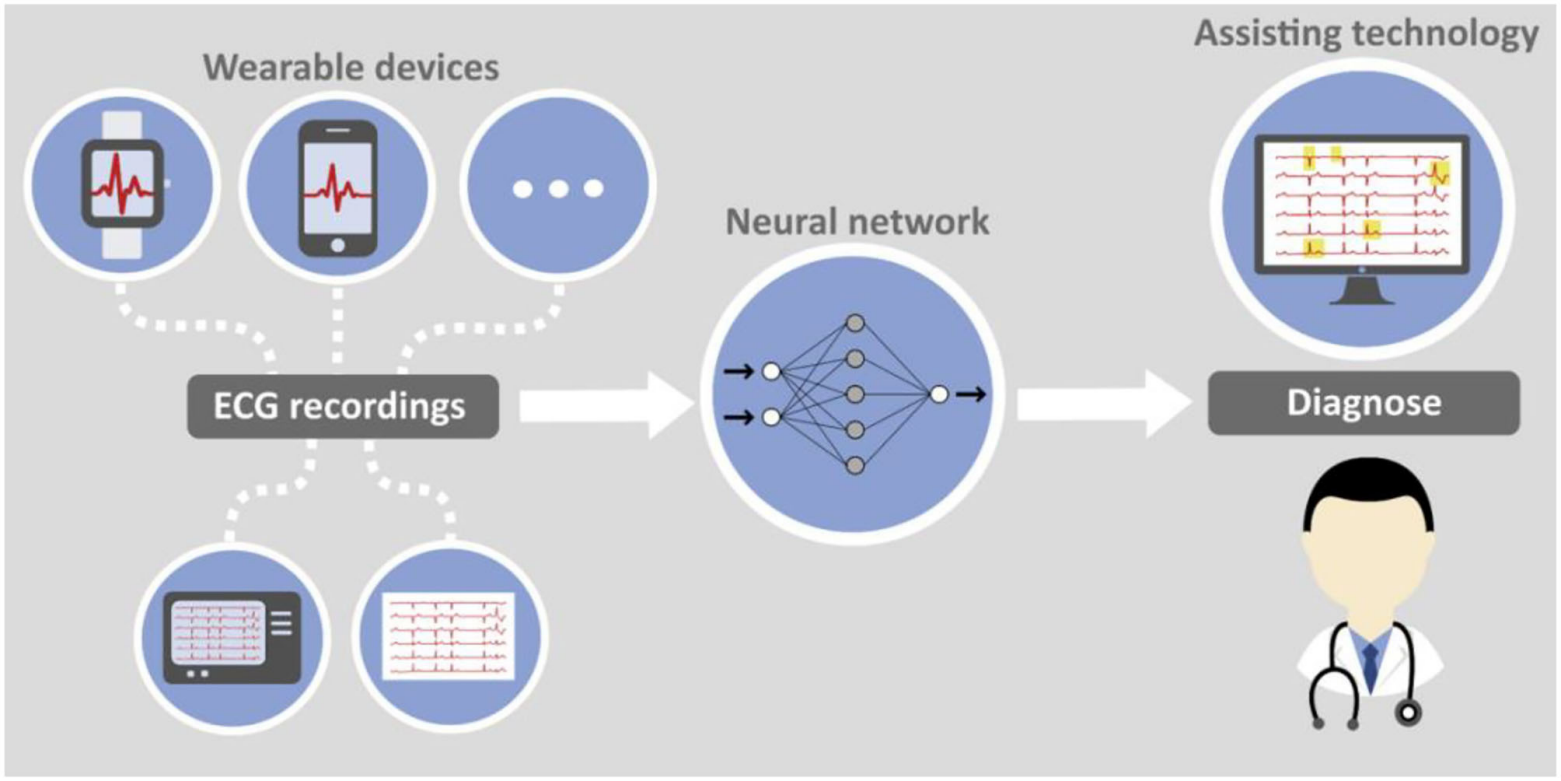
Computer-aided ECG analysis with doctor and assisting technology.

Figure 3 jointly presents both aspects of computer assisted ECG diagnosis with data either collected traditionally or with wearable devices.

### Medical Education

#### Medical Policy

Rheumatoid arthritis (RA) is a systemic autoimmune disease characterized by chronic inflammation and progressive joint destruction (40, 41). With a population prevalence of 1%, RA is the most prevalent inflammatory joint disease (42). Imaging plays a key role in diagnosis and disease monitoring. For instance, X-ray images of hands and feet are of central importance for the disease diagnostics and the assessment of disease progression. However, a variety of additional diagnostic imaging techniques are available and used depending on the particular question to answer (43). The appropriate use as well as the correct interpretation of imaging is complex and requires a sophisticated education and training.

#### Technological Solution

Advanced visualization and interactivity of individual 3-dimensional bone images can amend education in rheumatology. Virtual Reality (VR) provides a new access to the disease and its course through the interaction with a virtual patient and his or her medical history. Users of the VR technology act in a virtual and immersive environment that offers 3-dimensional anatomic and pathological images. This concept applies to students, specialists, physicians on advanced trainings, or scientists.

**Figure 4:**
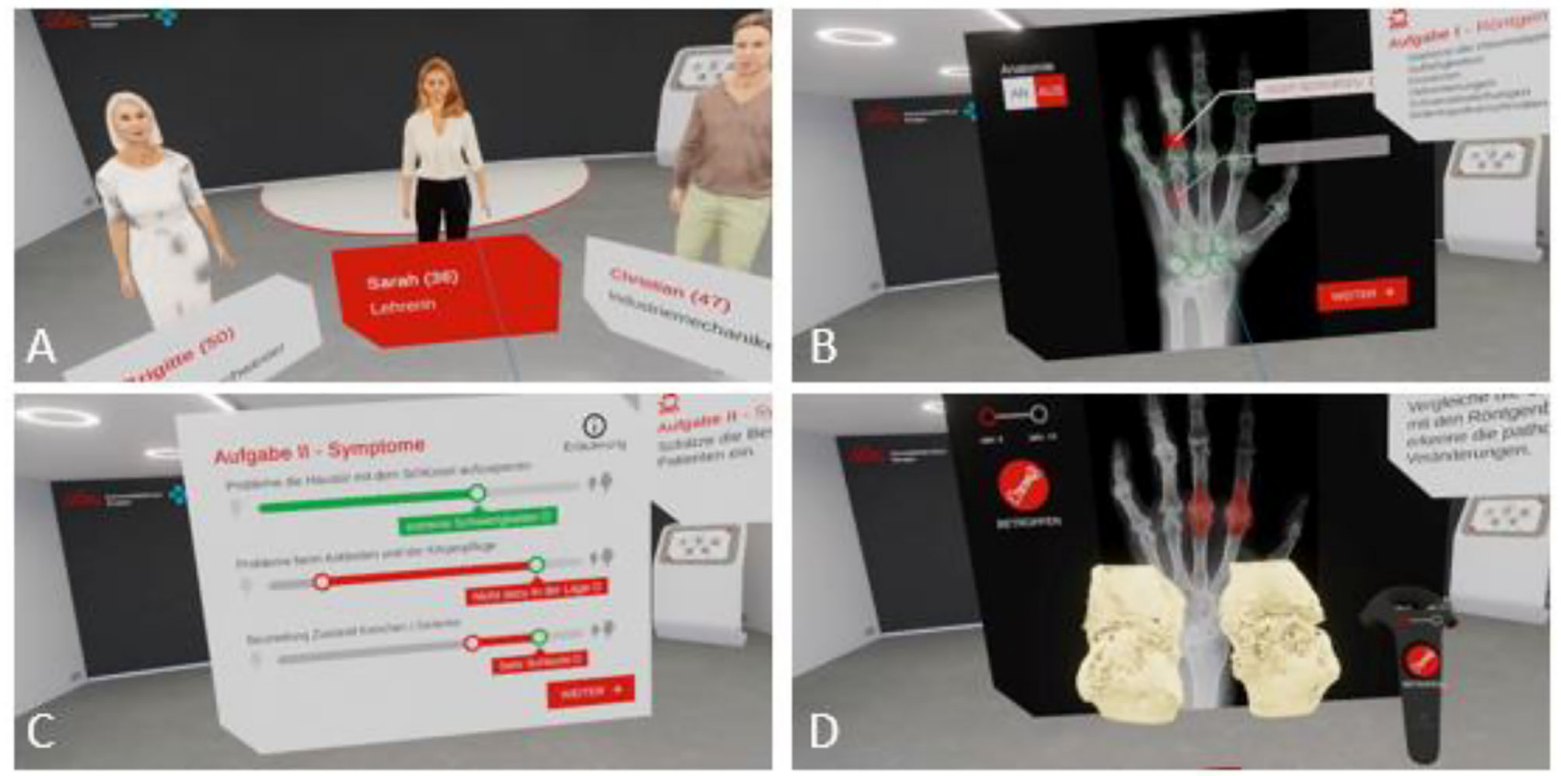
Rheumality-Virtual reality in medical education. Depicted is the welcome scene with the selection of three virtual patients with rheumatoid arthritis and psoriasis arthritis **(A)**. Users can evaluate the X-ray images of these patients for pathological arthritic changes **(B)** and assess the disease burden of the patients **(C)**. The immersion capability of the 3D visualization of arthritis joints allows in-depth inspection of specific disease-related changes **(D)**.

In 2016 the University Hospital of Erlangen and Lilly Germany established a project to connect VR with rheumatology (44) with the aim to improve the didactics of rheumatology education of doctors in training and medical students. A program, called Rheumality ©, was developed that converts 2-dimensional high-resolution computer tomographic data of finger and hand joints into a 3-dimensional VR images. These imaging data are combined with the corresponding patient history and clinical data. Compared with conventional imaging technologies, the VR model allows an immersive experience incorporating disease specific clinical aspects and characteristic pathologies, hence providing an effective learning system. Users of the VR platform can touch, scale or even immerge into typical RA pathologies such as erosions (Figure 4).

The program was positively received with 83% of the users assessed their virtual journey with the highest possible rating (44). Based on this positive feedback, the project was extended to other German medical schools such as the University of Gießen, where medical students can now learn to better understand diseases such as rheumatoid arthritis or psoriasis arthritis using this innovative VR program (45).

In medical education profound understanding of anatomical aspects is essential as a basis to adequately comprehend pathological changes. With a decline in anatomy courses (46) due to the change in requirements to medical education the need for other methods to communicate anatomical sciences grew. Several projects evaluated the use of VR in anatomy learning suggesting that integration of virtual-reality may facilitate knowledge transfer and increase study motivation (47). Nevertheless, in educational cases with need for individual feedback e.g., on physical actions a face-to-face training might still provide better results (48).

## Implications and Actionable Recommendations

Based on the use cases presented in the previous section, in this section we first discuss the implications (section Implications) for trust and trustworthiness of adding the new stakeholder of “technology provision and providers” to the Health 3.0 tetrahedron to create the Health 4.0 socio-technical ecosystem. On the basis of this discussion, we then derive a set of actionable recommendations (section Actionable Recommendations).

### Implications

Technology Developers enter the tetrahedron of trust in the center and possess vital relations to the traditional players of Health 3.0. The Health 3.0 trust matrix is extended with an additional row and column as depicted in Table 2. Yellow, green and red dots indicate relevance three use cases (respectively supervised exercise, ECG diagnosis and medical education). We focus on the perspective of the technology developers and neglect obvious effects that are subsumed in the traditional relations as previously presented in Table 1.

**Table 2:** Health 4.0 trust matrix, yellow green and red dots indicate implications for uses cases 1 (supervised exercise), 2 (ECG diagnosis) and 3 (medical education), respectively.

	**Medical practitioners**	**Patients and patient reps**	**Public health**	**Medical authorities**	**Technology developers**
Medical practitioners					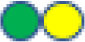
Patients and Patient reps					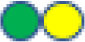
Public health	**Health 3.0**				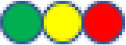
Medical authorities					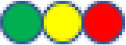
Technology developers	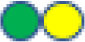	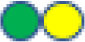	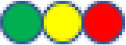	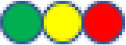	

Green and yellow dots represent use cases in which technical devices become part of the patient medical practitioner relation. One of a more diagnostic and one of a more therapeutical character. In both cases a basic requirement is proper and responsible use of the technology according to specifications on the basis of the assumption that design and implementation of devices and processes are fundamentally benevolent. Adequate and confidential use of data with proper anonymisation in case of further use is subsumed in this aspect. Obviously, technology developers must have trust in medical authorities that innovations are integrated into guidelines and are recommended if the tech-assisted treatment is better. Using complex technology to teach and train medical staff again requires trust in the principal correctness and a soundly evaluated didactical process. Public health and medical authorities trust in the efficacy of the innovative education.

In the following subsections, we consider these implications in more detail for each of the use cases.

#### Implications of Orbis App

From this use case, the following extended or side-effected trust relationships can be identified.

Firstly, healthcare practitioners trust the technology developer to “design for wellbeing.” The use of value-sensitive design (49) is recommended in order to put a qualitative human value, such as patient wellbeing, as a primary design requirement, alongside functional and non-functional requirements. Moreover, healthcare practitioners trust the patients to perform the prescribed programme of exercise. This is a variation of the medic's trust in a patient to complete a course of antibiotics, and while that is difficult to monitor, it is for this reason that ORBIS supports remote supervision as well as self-supervision.

The technology developers trust the medical practitioner to prescribe the app to the appropriate user group, i.e., the group for whom it has been designed. The history of procurement in software engineering is not good for COTS (commercial off-the-shelf) software designed for one purpose and user groups and being purchased and applied for another purpose and/or group. In addition, the technology developers trust the patients to register and record data accurately. The levels of distrust in social media are such that many people do not register with correct data, there is ample evidence of “cheating” using fitness apps (50). This creates two potential problems. Firstly, accurate data is required if the aggregated data is to be reliable for informing public health policy. Secondly, one of the key long-term properties of such systems is congruence, e.g., scaling up or down exercise challenges according to progress and rehabilitation. Moreover, machine learning algorithms will learn incorrect relationships if fed with biased data (51).

The patients trust the technology developers to continue to protect their privacy and the confidentiality of information that they reveal in a clinical setting. It is at this point that some of the requirements of the Hippocratic Oath extend from the healthcare provider to the technology developer, but the developer is under no such allegiance to a code of conduct. The patient trusts the doctor to make the best diagnosis, and may have expectations of being treated with a device as the most up-to-date treatment. Regrettably, in an age where convenience is almost a human virtue (52), many people expect to take a pill and be instantly cured, and do not see their health as a long-term “co-production,” i.e., they have to invest effort into maintaining their health in partnership with their healthcare providers.

The trust relationship between technology developers and public health authorities is complex but critical. At the root of this is the risk of aggregate data disappearing behind a corporate firewall: we need to ensure that medical assets (especially data) are available, accessible and affordable. (A prime example of this is parkrun (see https://www.parkrun.com), which collects huge amounts of personal data on a weekly basis, but makes access to the (anonymized) database freely available to researchers showing good cause. It is not clear that other fitness apps all do the same).

#### Implications of ECG Analysis With Deep Learning

This use case principally addresses two aspects of using technology for diagnosis of heart disease. One is the perspective of using deep learning for less invasive and less expensive diagnosis in a setting with ECG recordings traditionally taken in clinical environments. The other is the quite obvious generalization of AI applied to ECG recordings delivered from wearable devices.

The first requires medical practitioners to have trust in the correctness of the algorithm. Medical authorities eventually must integrate the use of the alternative method into their guidelines which will only be possible if technology developers give proof for the validity and soundness of their algorithms.

Adding the wearable devices to the picture, the patients now need to trust medical technology in form of a particular device (e.g., heart monitor, blood pressure monitor, oximeter, …), and the algorithm used for diagnosis in much the same way that they trust a medical professional.

For this relationship to work, patients need to trust that the device has been designed in their individual interests and the public interest at the core of the design, and that it truthfully functions according to what it is supposed to do. Additionally, the device must be trusted to keep data safe, secure and confidential (53); and that it is being used responsibly and not being sold off to third parties for some other purpose, e.g., surveillance or advertising. Medical professionals need to have this same trust in the technical device since usually they are those who recommend the use of it. Even from the perspective of the device trust is also required: the device needs real and accurate data, both from the patient (user) and from the medical professional to function as it was designed.

#### Implications of VR Use Case

The integration of VR technology opens a new way of medical education for students and physicians, but also introduces a potential disruption of trust. In a traditional academic teaching environment, there is a trust relationship between lecturer and student based on personal interaction and academic degrees assuring quality standards. With the introduction of VR technology in part replacing the lecturer leads to the need of students to trust technology trained by medical professionals but also by technology companies to provide adequate and evidence-based knowledge. There is also a further layer of trust concerning patients and their treating physician. Currently, patients assume that their personal physician was trained by experienced academic teachers with values like the ones written out within the Hippocratic oath as general objectives of providing health care. With technology taking over parts of knowledge mediation in medical education other interests might be part of the motivation behind the taught information.

### Actionable Recommendations

From the presentation of use cases and the implications discussed above, we would argue that trusting a technological device is essentially a proxy for trusting its creators, who are “new biotic factors” in the healthcare ecosystem but are generally one step removed from patients and medical professionals and without direct mutual contact with them. Therefore, trusting in a device basically implies trusting in the benevolence of the creators of the device. This is problematic as there is, as yet, no broadly accepted and applied equivalent to an obligation such as the Hippocratic oath for developers, who may have a financial incentive or commercial imperative which over-rides the values of trust, propriety and the “special relationship” between patients and practitioners enshrined in that oath.

Therefore, we propose the following nine actionable recommendations; for each we indicate which actor in Health 4.0 we believe should take the lead in taking action on the recommendation.

#### First

Verification and validation procedures for medical products (medical authorities) analogously to e.g., the Medical Device Regulation (MDR) already in place for specifically classified devices. A medical practitioner endeavors to ensure that no harm is done, and some good is done, to the patient. An extension of standard procedures in software engineering for products in the medical domain would be verification to ensure “nothing bad happens” and validation to ensure “something good happens”. These procedures can be administered by a trusted (sic) third party such as medical standards bodies to provide some kind of quality-assurance kitemark, similar to the terms of use regarding intellectual property and terms of service that have been proposed for privacy- and attention-enhancing technologies (54). Products that do not meet this standard can be treated as “alternative medicine.”

#### Second

A European (or ideally WHO approved) charter for green prescriptions and patient data protection (public health administration). Medical apps (like Orbis) being prescribed to treat conditions that respond to exercise but also promote sustainability should be promoted by trans-national organizations but should nevertheless be subject to the same licensing requirements, for example to avoid outcomes like the opiod crisis in the United States. Moreover, the use of apps necessarily implies the acquisition of data, and patients should be able to trust that the data is secure and only used beneficially and not mischieviously or dishonorably, either targeting individuals (e.g., for advertising, identification, life insurance premiums, etc.) or collectively [e.g., the national government of the UK trying to sell aggregated patient data to big tech (55)].

#### Third

Improved training provision for engineers in data, information and knowledge management (technology developers). While there is evidence that engineering courses in Higher Education take ethics more seriously, and there are ethical standards promoted by professional organizations such as ACM and IEEE, there is still scope for improvement. However, part of the problem is resistance from students themselves: many do not readily accept that is important for them to study and understand these issues and recognize their responsibilities. This actually suggests that critical thinking and civic education need to start earlier in the education process and be a formative part of that process (56).

#### Fourth

A new design methodology for public interest technology (technology developers). In recent years, as already mentioned, the design methodology of Value-Sensitive Design (VSD) has been proposed (49), to establish qualitative human values as “supra-functional” requirements beyond the functional and non-functional requirements specified by traditional software engineering methodologies. However, the design of medical products impacts on many values that are in the public interest–for example, privacy and data protection, public health, and properly-trained practitioners. For a matter of public interest as the value in question, a new methodology has been proposed (57) for developing technology in which this aspect of public interest is a primary design requirement, recognizing that this aspect can be (even) harder to operationalise than a qualitative value and even riskier to metricate or commodify. Design in the public interest needs to be led by developers but with respect to lived experience and lived expertise, and can only be most successfully operationalised if everyone impacted by Health 4.0 believes in the science, trusts in the technology (and each other), and cares enough to work together toward successful collective action.

#### Fifth

Encourage personal responsibility for well-being (patients and patient representatives). A common misinterpretation of the right to health (or the right to access healthcare at the time and point of need) is to defer responsibility to a “healthgiver” and demand the prescription of a pill that will provide an instant “fix.” Given the way that people defer to voice-activated virtual assistants in place of cerebral activity, it is also necessary to counter the potential *over-trust* in technology fuelled by people's well-documented automation bias. Therefore, related to the issue of public interest technology, there needs to be public information programmes which reinforce the idea that, like education, health is a co-production, and relies on contributions from two parties (i.e., student-teacher in education, patient-practitioner in healthcare), but which also re-affirm the authority of the doctors and mutual agreement on the facts (58).

#### Sixth

Provide systems of accountability for malpractice (medical authorities). A recurring problem of machine learning (especially deep learning) is that the algorithms work, but there is no clear explanation as to why they work. While some are convinced that, e.g. algorithms for protein folding, will fundamentally change evidence-based medicine, there is risk that this will undermine respect for the scientific method itself (59). This runs the further risk of a loss of accountability seen, for example, in lethal autonomous weapons, which lets everyone off the hook when use of drones goes wrong and attacks a civilian target by mistake: the president has not done it, was the general who gave the order; although the general has not done it, it was the soldier operating the drone; but the soldier has not done it, it was the programmer; and the programmer has not done it, it was the algorithm. Therefore some link has to be established between a medical product and its provenance in case of malpractice, inadvertent or otherwise.

#### Seventh

No short-cuts or compromises of care-giving in pursuit of profit-seeking (medical practitioners). Faced with a massive national problem presented by the recent surge of mental health problems, especially amongst young people, the appeal of, for example, chatbots as surrogate therapists or counselors is strong. However, the risk here is that sentiment analysis can show how the mind can be manipulated for profit, not how it can better readjust to the complexity of a fast-moving world, uncertainty in an unprecedented situation like the COVID-19 pandemic, or managing grief after an unexpected loss. It needs to be understood that such technology can only be a complement to human interaction, not a replacement for it, and that mental health programmes need to be properly funded (60).

#### Eighth

Compulsory review to avoid bias and entrenchment in medical training (medical practitioners). A fundamental and essential characteristic of Health 1.0-3.0 has been systemic self-improvement: as new evidence has been made available, so medical practice and procedures have improved; this in turn has made more evidence available, and the process of self-improvement repeats. A potential problem in medical training is to use biased datasets, bias being a critical problem in machine learning (51) (a common refrain in computer programming was “garbage in, garbage out;” in machine learning it should be “bias in, bias out”). However, a potential further problem is not just training students on biased data, but training students on biased and *out-dated* data. Therefore there should be compulsory review of all datasets being used in medical training to ensure that the datasets being used are both unbiased and “fresh.”

#### Ninth

Guidelines to ensure adherence to scientific evidence and best medical practice (public health administration). The development of the ORBIS app was made with clear reference to the NICE guidelines and pilot studies which demonstrated the effectiveness of the intervention (as well as best practice in data acquisition and information processing with respect to privacy). It has to be ensured that medical products are grounded on the current scientific body of evidence regarding adequate data as well as medical outcome measures and the integration into clinically relevant diagnostic and therapeutic algorithms. Following accepted medical guidelines recommended by the relevant professional associations of the respective region and culture group could provide the needed confidence to build trust in the technological product.

## Discussion and Conclusions

In conclusion, we have demonstrated that trust is the cornerstone of healthcare, and absolutely critical to the well-being itself of the Health 4.0 ecosystem. However, trust in this new ecosystem is vulnerable in a number of ways. It is for instance threatened by influential stakeholders from inside or outside of the system: government bodies as part of public health may be overly influenced by interest groups to prevent legislation going through, such as for sugar taxes, or general practitioners can fall prey to biased marketing actions on behalf of pharmaceutical companies: the opioid crisis is an example for that. Lack of education of patients about medical procedures is another gateway for harm, such as the misconception that antibiotics can remedy mostly any symptom and disease or that traditional or alternative therapies are more or at least equally effective as modern medicines for specific diseases.

However, the key issue is that, inside the traditional healthcare system, most of the participants are humans: individuals who have either been medically trained to adhere to the Hippocratic oath, or who have the principal understanding to appropriately interpret it in various situations they may come across. With technology this would mean explaining this oath to a technology developer who is sufficiently removed from the patient to have conflicting priorities in terms of technological development, vested economic interests, and data security as they are beholden to their shareholders.

Thus the information flowing from the public health bodies to the practitioners and vice versa is no longer a direct one. Instead it now also flows to the technology developers. The developers use data from public health to create products which provide medical practitioners with more information than ever before. Diagnoses and the state of health of individuals can now be interpreted with potentially greater accuracy and thus better inform public health. This change can have far reaching consequences. In an ideal world this information would flow unimpeded, however in reality there may be a firewall between the data collected from medical practitioners and that which is reported back to them. This may be because the technology created is unable (either due to technological limitations or security concerns) to share or collect relevant data. Even worse, the creators of technology could be unwilling (due to proprietary data concerns) to do so.

In cases in which data is not being appropriately shared for the greater good, and the feedback loop is incomplete, trust in the system could decline and as a result its efficacy degrade. The other case is more serious, instead of not sharing the data, the data could be sold for profit, turning the patients into revenue streams and exploiting potentially vulnerable individuals for financial gain. In this latter case, complete loss of trust in the device and hence the medical professional who recommended it, could occur.

Similarly, some patients can re-attach the trust they had in their personal physician to a website, which might be designed merely to monetise traffic, or, in age of misinformation and disinformation, might have some other unscrupulous agenda. The proliferation of the anti-vax movement is attributed to a substantive online presence (61) spreading misinformation by masquerading as expert opinion (qualifications of sources and provenance of data not being verified). This has already led to a reduction in the overall number of children being vaccinated and outbreaks of diseases commonly associated with childhood, but presents a particular risk to public health of all generations in the midst of a pandemic. Clearly, social media with its negative reinforcement (a loss of trust in part of the system leads to a loss of trust in the whole) shows how deeply new technology can have an adverse impact on the medical ecosystem.

In final conclusion, we remark that medical policies and guidelines address the full spectrum of medical professionals who follow the recommendations producing stability and continuity in the processes of the health system. More comprehensive use of intelligent technical equipment for patients beyond fitness-motivated and technically-affinitive groups raise new questions of trust and reliability.

Guidelines are directives and are generally not legally binding. This means that doctors can deviate from the treatment recommended in the guideline if they think that in a particular case it is less suitable for the patient than an alternative diagnostic means or therapy. However, such deviations have to be well justified in each case^1^.

Integration of Health 4.0 will over time gradually become an integral part of guidelines and policies. The scientific community has to provide data and evidence that implementation of Health 4.0 is feasible, safe and beneficial. Only through a high degree of evidence supported by reliable prospective studies can trust in technology and medical devices be established and sustained.

## Author Contributions

MG, JH, KM, TK, and JP contributed to the conception or design of the work. Data acquisition, analysis, and/or interpretation of data for the use cases was contributed by KM and JP regarding the ORBIS use case (Smartphone Assistance intermittent claudication patients) by DS, AK, TW, and DE regarding the VR use case (Virtual Rheumatology) and by MG, JH, and TK regarding the third use case (ECG diagnosis with Deep Learning Algorithms). MG, KM, JH, TK, and JP drafted the manuscript and all authors critically revised the manuscript. All authors gave final approval and agree to be accountable for all aspects of the work ensuring integrity and accuracy. All authors contributed to the article and approved the submitted version.

## Funding

MG, TK, and JH were supported by Research Campus of Central Hessen (RCCH).

## Conflict of Interest

DE and TW are employed by Eli Lilly Germany. The remaining authors declare that the research was conducted in the absence of any commercial or financial relationships that could be construed as a potential conflict of interest.

## Publisher's Note

All claims expressed in this article are solely those of the authors and do not necessarily represent those of their affiliated organizations, or those of the publisher, the editors and the reviewers. Any product that may be evaluated in this article, or claim that may be made by its manufacturer, is not guaranteed or endorsed by the publisher.

## Footnotes

^1^https://www.gesundheitsinformation.de/was-sind-leitlinien.html (accessed November 1, 2021).
